# Choosing Wisely Canada rhinology recommendations

**DOI:** 10.1186/s40463-020-00406-9

**Published:** 2020-02-28

**Authors:** Neil Arnstead, Yvonne Chan, Shaun Kilty, Ragavan Ganeshathasan, Armin Rahmani, Eric Monteiro, Rhinology Subspecialty Group of the Canadian Society of Otolaryngology – Head & Neck Surgery

**Affiliations:** 1 Department of Otolaryngology – Head & Neck Surgery, University of Toronto, C. David Naylor Building, Suite 120, 6 Queen’s Park Crescent West, Toronto, ON M5S 3H2 Canada; 2 Department of Otolaryngology – Head & Neck Surgery, University of Toronto, Trillium Health Partners, Suite 102-101 Queensway West, Missisauga, ON L5B 2P7 Canada; 3 Department of Otolaryngology – Head & Neck Surgery, University of Ottawa, The Ottawa Hospital Civic Campus, 737 Parkdale Avenue, Room 259, Ottawa, ON K1Y 1J8 Canada; 4 Department of Family Medicine, University of Western Ontario, Western Centre for Public Health and Family Medicine, 1465 Richmond Street, London, ON N6G 2M1 Canada; 5 Department of Psychiatry, University of Toronto, 250 College Street, 8th floor, Toronto, ON M5T 1R8 Canada; 6 Department of Otolaryngology – Head & Neck Surgery, University of Toronto, Mount Sinai Hospital, 600 University Avenue, Suite 404, Toronto, ON M5G 1X5 Canada

**Keywords:** Choosing Wisely Canada, Medical overuse, Sinusitis, Nasal fracture, Rhinology, Anti-bacterial agents, Drug utilization, Computed tomography, Radiography

## Abstract

The Choosing Wisely Canada campaign is an initiative that aims to involve physicians and patients in collaborative decision making to avoid unnecessary tests and treatments. The Rhinology Subspecialty Group of the Canadian Society of Otolaryngology – Head & Neck Surgery developed a list of five evidence-based recommendations for the management of acute rhinosinusitis and nasal fractures: (1) don’t prescribe antibiotics to patients with acute sinusitis who do not meet the diagnostic criteria for acute bacterial rhinosinusitis; (2) don’t order a CT scan for uncomplicated acute rhinosinusitis; (3) don’t order plain film sinus x-rays; (4) don’t swab the nasal cavity as part of the work up for rhinosinusitis; and (5) don’t order a plain film x-ray in the evaluation of nasal fractures.

## Introduction

The Choosing Wisely Canada campaign is an initiative that aims to involve physicians and patients in collaborative decision making to avoid unnecessary tests and treatments. The Canadian Society of Otolaryngology – Head and Neck Surgery (CSOHNS) is a proud partner in this effort, and has previously released recommendations to guide investigations in Otology/Neurotology [1] and Head and Neck Surgery [2]. The CSOHNS is dedicated to improving patient care through scientific research, patient and physician education, and the maintenance of the highest professional standards.

## Methods

The Rhinology Subspecialty Group of the CSOHNS, representing national leaders within the subspecialty, were asked to create a list of recommendations for unnecessary tests or interventions that were commonly performed. The supporting evidence for each candidate recommendation was then reviewed and summarized for panel review. During a second face-to-face meeting, the candidate recommendations were reviewed and the Subspecialty Group voted on a final set of five recommendations. The final version of the list was then circulated and approved by members of the Rhinology Subspecialty Group. Choosing Wisely Canada groups across multiple specialties reviewed and refined the consensus recommendations [3].

### Recommendations

#### Do not prescribe antibiotics to patients who do not meet the diagnostic criteria for acute or chronic rhinosinusitis

The prevalence of a bacterial infection during acute rhinosinusitis is estimated to be 2–10%, whereas viral causes account for 90–98% [4]. Despite this, 82% of Canadian patients diagnosed with acute sinusitis received a prescription for antibiotics [5]. Differentiating viral rhinosinusitis from acute bacterial rhinosinusitis (ABRS) is challenging because the symptoms are overlapping, but is critical to avoid inappropriate antibiotic prescriptions.

ABRS is diagnosed when (a) symptoms persist beyond 7–10 days without improvement, or there is a worsening of symptoms after 5–7 days after an initial improvement *AND* (b) the patient has at least two of four of the following symptoms: facial Pain/pressure/fullness, nasal Obstruction, nasal purulence/discoloured postnasal Discharge, decreased/absent Smell (Table 1).

**Table 1:** ABRS diagnosis requires the presence of at least 2 of the following symptoms^a^

	Symptom
P	Facial **P**ain/pressure/fullness
O	Nasal **O**bstruction
D	Purulent/discoloured nasal or postnasal **D**ischarge
S	Hyposmia/anosmia (**S**mell)

^a^At least 1 symptom must be nasal obstruction or nasal purulence/discoloured postnasal discharge. Thus, a diagnosis requires at least two PODS, one of which must be O or D

Consider ABRS when symptoms persist beyond 7 to 10 days, or worsens after 5 to 7 days following an initial improvement (“double worsening”)

In patients who meet the criteria for ABRS with mild (occasional, limited episode) or moderate symptoms (steady but easily tolerated), intranasal corticosteroid sprays alone are often sufficient. Antibiotics can be considered for patients with severe symptoms (hard to tolerate, interfering with activity or sleep) or those who fail a 72 h trial of intranasal corticosteroids after the diagnosis of ABRS has been made [6–9].

Management of viral rhinosinusitis is primarily focused on symptomatic relief. Antibiotics are ineffective for viral illness and do not provide direct symptom relief. The drawbacks of antibiotics include allergic reactions, potential drug-drug interactions, increased costs, and increased bacterial resistance. Symptomatic treatment can include analgesics, nasal saline rinses, intranasal corticosteroids, oral or topical decongestants, and mucolytics [6, 10].

#### Do not order a CT scan for uncomplicated acute rhinosinusitis

Radiographic imaging for patients presenting with uncomplicated acute rhinosinusitis is not recommended, unless a complication or alternative diagnosis is suspected [6, 10, 11]. A sinus CT scan is a highly sensitive test for rhinosinusitis, and a normal study confidently rules out active sinusitis of any etiology. However, abnormal sinus CT imaging findings, including air-fluid levels, mucosal thickening, and complete sinus opacification, are nonspecific and can be seen with bacterial or viral sinusitis, as well as in up to 42% of asymptomatic healthy individuals [6]. In a prospective study of healthy young adults experiencing a new cold, CT scans showed that 87% of the subjects had significant abnormalities of their maxillary sinuses [12]. Therefore in acute rhinosinusitis, a CT scan has minimal utility because its findings are not specific to a diagnosis of acute rhinosinusitis, and does not help guide the need for antibiotics since it cannot reliably distinguish viral from bacterial disease. Consider CT imaging when a complication of ABRS is suspected based on severe headache, altered mental status, facial swelling, cranial nerve palsies, proptosis of the eye, or other clinical findings [10].

#### Do not order plain film sinus x-rays in the work-up of rhinosinusitis

Plain film x-rays of the sinuses should not be ordered in the work-up of sinusitis [6, 13]. Plain films have poor sensitivity (25–80% compared to CT scan) and they cannot be relied upon to confirm or reject the diagnosis of either acute or chronic sinusitis [14]. Findings such as air-fluid levels and complete sinus opacification are present in only 60% of cases of rhinosinusitis, and cannot differentiate between viral and bacterial etiologies [15]. The complicated anatomy of the ethmoid sinuses and critical sinus drainage pathways are not delineated effectively with plain films, and are inadequate for operative planning. Given that the findings of a sinus x-ray cannot be relied upon to diagnose rhinosinusitis, guide antibiotic prescribing, or plan surgery, they do not provide value in patient care and should be avoided. If imaging is indicated, a CT scan of the sinuses is the preferred initial radiographic modality, while MRI is generally reserved for assessing for intracranial complications, as well as in the setting of other diagnoses, such as intracranial tumours [6, 10, 11].

#### Do not swab the nasal cavity as part of the work up for rhinosinusitis

Acute bacterial rhinosinusitis is a clinical diagnosis that does not require proof of a culture-identified pathogen. When patients meet criteria for uncomplicated ABRS, empiric antibiotic selection should be based on typical causative pathogens (i.e. *Streptococcus pneumoniae, Hemophilus influenza, Moraxella catarrhalis,* and *Staphylococcus aureus*), local bacterial resistance patterns, and patient factors (e.g. risk of exposure to penicillin-resistant *S. pneumoniae* in daycare or healthcare settings*,* allergy to penicillins, age, or immunosuppression putting patients at higher risk of infectious complications) [11]. Nasal swabs are contaminated by normal nasal flora and results correlate poorly with causative pathogens in rhnosinusitis [11]. In many hospitals, a nasal swab will be processed to only report on the presence or absence of *S. aureus*, rather than a full culture for speciation. In situations where cultures are required, such as intraorbital or intracranial complications, endoscopically-guided culture of the middle meatus or a maxillary sinus aspirate are the preferred methods for obtaining samples of the causative pathogen [6].

#### Do not order a plain film X-ray in the work-up of nasal fractures

Plain film x-rays should not be ordered as part of the management of nasal fractures. The decision to reduce a nasal fracture depends on numerous factors including patient preference, external deformity, and breathing difficulty, none of which are effectively assessed by an x-ray. They have a very low sensitivity and specificity, with 63.3 and 55.7% respectively [16]. As such, plain x-rays are unable to accurately diagnose occult fractures. Despite being commonly ordered for medicolegal documentation of nasal fractures, the poor sensitivity and specificity brings into question their value in medicolegal proceedings [17, 18]. In studied cohorts, no unsuspected facial fractures were identified solely on nasal x-rays [19], and no negative effects on management occurred when an institution instituted a “no nasal x-ray policy” [20]. Nasal fractures may occur simultaneously with other facial fractures, and a CT-scan of the facial bones is appropriate if facial fractures are suspected based on history and clinical examination findings such as telecanthus, palpable step deformity, crepitus on palpation, ocular symptoms/signs, malocclusion, raccoon eyes, collapse of the nasal dorsum, or penetrating injuries. Overall, nasal x-rays do not contribute to diagnosis, documentation, or management decisions, and should not be ordered.

## Discussion

These recommendations are not intended to be used to establish payment or insurance coverage decisions. Rather, they are meant to spur collaborative conversations among both physicians and patients about what is appropriate and necessary in the care of rhinosinusitis and nasal fractures. Each patient situation is unique, so physicians and patients could use these recommendations to design an appropriate treatment plan together.

## Conclusion

Otolaryngologists are frequently involved in the workup and management of patients with these common conditions. It is important to recognize inappropriate tests and treatments. Otolaryngologists are well positioned to work in education and advocacy to bring this information to patients and other physicians.

## Data Availability

No original data was used in generating these recommendations – primary data would be sourced from the cited sources.

## Abbreviations

ABRS: Acute bacterial rhinosinusitis
CSOHNS: Canadian Society of Otolaryngology – Head & Neck Surgery
CT: Computed tomography
URTI: Upper respiratory tract infection
